# Supplementary material for: Complexome profiling of the Chlamydomonas psb28 mutant reveals TEF5 as an early PSII assembly factor
Source: Plant Cell. 2025 Mar 17;37(6):koaf055. doi: 10.1093/plcell/koaf055 (PMC12164590; doi:10.1093/plcell/koaf055)
Supplement: koaf055_Supplementary_Data [file koaf055_supplementary_data.zip › psb28_interactive.zip!/plots/intensity/qualityPlots/peptides/atpI_Cre-1.g2716959.t1.p1.html]

### Cre-1.g2716959.t1.p1

Trivial name: atpI  
SeqCoverage [median]: 15.10  
Mass: 26.18 kDa  
  
 

| Sequence | MissedCleavage | Mass [Da] | wtIntensity | muIntensity | Score |
| --- | --- | --- | --- | --- | --- |
| GLGYFSR | 0 | 798.40 | 271862.38 | 246304.34 | 151.17 |
| KGLGYFSR | 1 | 926.50 | 55561.21 | 34099.61 | 214.25 |
| STPDGFQNFTELVTEFIR | 0 | 2100.02 | 28701.98 | 10612.09 | 161.21 |
| TQIGEEDYLK | 0 | 1194.58 | 151870.87 | 126516.25 | 227.27 |
|  |  |  | 507996.4 | 417532.3 |  |
